# Availability and affordability of priority life-saving medicines for under-five children in health facilities of Tigray region, northern Ethiopia

**DOI:** 10.1186/s12884-018-2109-2

**Published:** 2018-11-29

**Authors:** Solomon Abrha, Ebisa Tadesse, Tesfay Mehari Atey, Fantahun Molla, Wondim Melkam, Birhanetensay Masresha, Solomon Gashaw, Abrham Wondimu

**Affiliations:** 1 Department of Pharmaceutics, School of Pharmacy, College of Health Sciences, Mekelle University, Mekelle, Tigray Ethiopia; 2 Clinical Pharmacy Unit, School of Pharmacy, College of Health Sciences, Mekelle University, Mekelle, Tigray Ethiopia; 3 Department of Pharmacology and Toxicology, School of Pharmacy, College of Health Sciences, Mekelle University, Mekelle, Tigray Ethiopia; 4 Department of Pharmacy, College of Health Sciences, Bahir Dar University, Bahir Dar, Amhara Ethiopia

**Keywords:** Priority life-saving medicines, Availability, Medicine Price, Affordability, Under-five children, Tigray Ethiopia

## Abstract

**Background:**

In developing countries, child health outcomes are influenced by the non-availability of priority life-saving medicines at public sector health facilities and non-affordability of medicines at private medicine outlets. This study aimed to assess availability, price components and affordability of priority life-saving medicines for under-five children in Tigray region, Northern Ethiopia.

**Methods:**

A cross-sectional study was conducted in Tigray region from December 2015 to July 2016 using a standard method developed by the World Health Organization and Health Action International (WHO/HAI). Data on the availability and price of 27 priority life-saving medicines were collected from 31 public and 10 private sectors. Availability and prices were expressed in percent and median price ratios (MPRs), respectively. Affordability was reported in terms of the daily wage of the lowest-paid unskilled government worker.

**Results:**

The overall availability of priority life-saving drugs in this study was low (34.1%). The average availabilities of all surveyed medicines in public and private sectors were 41.9 and 31.5%, respectively. The overall availability of medicines for malaria was found to be poor with average values of 29.3% for artemisinin combination therapy tablet, 19.5% for artesunate injection and 0% for rectal artesunate. Whereas, the availability of oral rehydration salt (ORS) and zinc sulphate dispersible tablets for the treatment of diarrhea was moderately high (90% for ORS and 82% for zinc sulphate). Medicines for pneumonia showed an overall percent availability in the range of 0% (ampicillin 250 mg and 1 g powder for injection and oxygen medicinal gas) to 100% (amoxicillin 500 mg capsule). The MPRs of 12 lowest price generic medicines were 1.5 and 2.7 times higher than the international reference prices (IRPs) for the private and public sectors, respectively. About 30% of priority life-saving medicines in the public sector and 50% of them in the private sector demanded above a single daily wages to purchase the standard treatment of the prevalent diseases of children.

**Conclusions:**

The lower availability, high price and low affordability of lowest price generic priority life-saving medicines in public and private sectors reflect a failure to implement the health policy on priority life-saving medicines in the region.

## Introduction

Being a critical improvement indicator of health, socioeconomic status and quality of life of a given population, there has been a global drive to improve the under-five children mortality rate during the past three decades [1]. To that end, over the past fifteen years, under-five global mortality rate has declined from 90.6 deaths per 1000 live births in 1990 to 42.5 in 2015 [2]. However, high rate remains in sub-Saharan Africa where one child in 12 dies before his or her fifth birthday – far higher than the average ratio of 1 in 147 in high-income countries [3]. In Ethiopia, children under-five mortality rate was reported to be 67 per 1000 live births in 2015, with one in every fifteen children dying before their fifth birthday [4].

According to the United Nations (UN) commission on life-saving commodities for women and children, many of these deaths are due to conditions such as pneumonia, diarrhea and malaria, which could easily be prevented or treated by simple and affordable medicines administered before, during and immediately after birth [5]. For instance, of the 6.3 million under-five deaths in 2013, around 15, 11 and 7% of them were caused by pneumonia, diarrhea and malaria, respectively [6]. Yet, early diagnosis and treatment with simple antibiotics could avert as many as 600,000 of deaths in case of pneumonia whereas, improving access to ORS would save as many of 1.3 million children who are dying annually from diarrhea [3, 7].

In 2011, the WHO departments of essential medicines and health products and other stakeholders developed a list of priority life-saving medicines for women and children with the main aim of supporting countries to plummet maternal, newborn and child morbidity and mortality [8]. According to this document, priority life-saving medicines are medicines which have the potential to save lives of children that should be available in all health systems and at all times. Medicines for the management of pneumonia, diarrhea, malaria, neonatal sepsis, HIV, vitamin A deficiency, tuberculosis and pediatric palliative care have been included under priority life-saving medicines for children’s health [8, 9].

Albeit some availability and affordability surveys [10, 11] have been conducted for children and adult on essential medicines, to the best of our knowledge, there was no a single previous study carried out to assess availability and affordability of the WHO recommended pediatric priority life-saving medicines in Ethiopia. Hence, the main aim of this study was to examine the availability and affordability of the life-saving priority medicines for children under five-years old in health facilities found in Tigray Region, Northern Ethiopia.

## Methods

A cross-sectional study was conducted in Tigray region, Ethiopia from December 2015 to July 2016. Tigray region lies in the Northern part of Ethiopia. It borders Eritrea to the north and Sudan to the west, while in the east it shares a regional border with the Afar region and in the south-west with Amhara region. The region is sub-divided into 7 administrative zones and 52 districts with its capital in Mekelle. The region has a population of 4,314,456, of which 630,862 are children under five years of age as per 2007 national census [12]. The health care system in the region comprises of tertiary and referral hospitals, zonal hospitals, district hospitals, health centers and health posts.

### Selection of health care facilities

The medicine outlets were selected using the WHO/HAI methodology, which has been validated to select a representative sample [13]. First, the main public hospital in the region, which is found in the capital city, Mekelle, was selected. Four public and five private medicine outlets (for example, hospital outpatient medicine outlets, dispensaries) which were in close proximity to the main public hospital were then randomly selected. Finally, additional private and public medicine outlets were chosen from other five survey areas within a 3-h drive from the main hospital. Accordingly, a total of 43 (10 public, 31 private and 2 nonprofit) medicine outlets were included in the study.

### Selection of medicines

All the medicines surveyed (except paracetamol suppository) in our study were identified from the list of “Priority life-saving medicines for women and children” developed by WHO in 2012 [8]. WHO had chosen the medicines on this list according to the global burden of the diseases and the evidence of efficacy and safety for preventing or treating major causes of maternal, newborn and child mortality and morbidity. The recommended priority life-saving medicines for children under-five are artemisinin combination therapy, rectal artesunate and artesunate injectable for malaria; zinc sulphate dispersible tablets and ORS sachets for diarrhea; amoxicillin (capsule), ampicillin ceftriaxone and gentamycin (powder for injections) for treatment of pneumonia; and ampicillin and procaine benzyl penicillin (powder for injections) for neonatal sepsis.

A total of 27 medicines, all of which registered in the country, were studied. For each medicine in the survey, data on price and availability were collected for the originator brand (OB) and lowest priced generic (LPG) at each facility. However, only availability data were collected for drugs such as antimalaria medicines, vitamin A, magnesium sulfate, calcium gluconate and zinc since these medicines in public sector are free of charge to all. The OB product was defined as a single-brand product marketed by the originator pharmaceutical company whereas “LPG equivalents” were defined as the same product sold under the generic name with the lowest unit price at each medicine outlet at the time of data collection in the survey [13].

### Data collection and analysis

Using a standard data collection format, data on availability and price were collected by well-trained pharmacists from March to May 2016. The principal investigator together with supervisors supervised the data collection process by checking all forms at the end of each day of the data collection. As part of the training workshop, a pilot test was conducted at retail medicine outlets, which could not form part of the survey sample but used to customize the data collection format. Data were coded, checked for completeness, consistency and accuracy for each medicine’s unit price and analyzed using Microsoft® Excel and Statistical Package for Social Scientists (SPSS® 20.0) statistical software. Data on public and private sectors were analyzed separately whereas data on non-profit sectors were not amenable to analysis as there were only two non-profit sectors found during this study and in order to be considered for analysis the number of non-profit facilities should be four according to the WHO/HAI methodology [13].

The analyses of the study focused on three measures: medicine availability, prices and affordability. Medicine availability was reported as the percentage (%) availability of an individual medicine at the surveyed outlets on the date of data collection [13]. Mean availability refers to the overall “basket” of medicines surveyed. To express the availability of medicines in the healthcare facilities, the following ranges were used [14]: < 30%, 30–49%, 50–80%, and > 80% for a very low, low, fairly high, and high availability, respectively. Prices were presented as MPRs, which are the ratios of the median local unit prices of medicines across facilities divided by their median IRPs [13]. The medicine prices obtained from the 2015 Drug Prices Guide issued by Management Science for Health (MSH) organization were adopted as the IRPs for core medicines [15]. MPR for a given medicine was calculated only if the medicines was available at a minimum of four facilities as per the WHO/HAI methodology and they were used as indicator to establish comparisons among countries. To calculate MPR, local median prices were converted to United States dollar (USD) using the exchange rate of commercial bank of Ethiopia at a buying rate of 21.8356 Ethiopian Birr (ETB) per 1 USD on the first day of data collection [16].

Notwithstanding the fact that there are no strict rules to interpret MPRs of medicines from medicine outlets, different studies [14, 17] reported using WHO/HAI methodology to explain the MPRs. Accordingly, if the MPR is twice of the IRP for a generic equivalent product, it can be considered as a cause for concern since the price is likely to be unaffordable [14]. In this study, the following MPR cut-off points: MPR ≤1.5 for public hospital patient prices and MPR ≤ 2 for retail pharmacies patient prices were used to represent acceptable local price ratios [18].

Affordability was estimated in this study as the number of daily wages of the lowest-paid unskilled government worker required to cover for the complete course of standard treatments of the selected diseases [13, 19, 20]. This was done by first computing the daily wage of the workers at the time of data collection, which was found to be 21.67 ETB per day (0.99 USD) [21]. The total costs of medicine for the complete duration of treatments of each disease were then determined and converted to the daily wages. Medicines that costed less than a day wage were considered affordable and those medicines with the cost of ≥ a day wage were considered unaffordable [22]. The total dose required to treat a particular health condition of children was calculated based on dose per kilogram (kg) method using 14.5 kg as the average weight for a 5 -year-old child in Ethiopia [21, 23].

## Results

### Availability of the priority life-saving medicines for children under-five

**Fig. 1:**
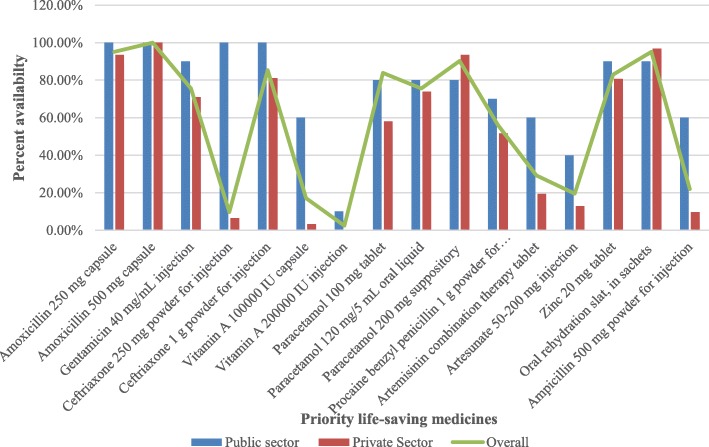
Availability of the selected priority life-saving medicine. Blue colored graphs represent availability of medicines in public sectors; Red colored graphs represent availability of medicines in private sectors and Purple colored line shows the overall availability of surveyed medicines

The overall availability of priority life-saving drugs for children under-five in this study was 34.1%. The average availabilities of all surveyed medicines in public and private sectors were 41.9 and 31.5%, respectively. The availability of medicines was found to vary with the type of medicine and sector. For instance, the availability of all life-saving medications in public outlets was higher than private sector except for paracetamol 200 mg suppository and ORS. Besides, the availability of artemisinin combination therapy for injection was almost thrice in the public sector compared to the private sector. Another important finding revealed in this study was the absence of morphine at any dose and dosage form, artesunate 50–200 mg suppository, paracetamol 200 mg suppository, oxygen medicinal gas, ampicillin 250 mg powder for injection and ampicillin 1 g powder for injection in both sectors (Fig. 1, Table 1). Moreover, there were no innovator brand medicines found in the public and private sectors.

**Table 1:** Availability of priority life-saving medicines for children under-five in selected health sectors found in Northern Ethiopia, 2016

Name of the medicine	Percentage (%) of medicine outlets where medicine were found		
	Public sector (10 outlets)	Private sectors (31 outlets)	Overall (41 outlets)
Amoxicillin 250 mg capsule	100.0	93.5	95.1
Amoxicillin 500 mg capsule	100.0	100.0	100.0
Gentamicin 40 mg/mL injection	90.0	71.0	75.6
Ceftriaxone 250 mg powder for injection	100.0	6.5	9.8
Ceftriaxone 1 g powder for injection	100.0	81.0	85.3
Morphine 100 mg granules capsule	0.0	0.0	0.0
Morphine 60 mg granules capsule	0.0	0.0	0.0
Morphine 30 mg granules capsule	0.0	0.0	0.0
Morphine 20 mg granules capsule	0.0	0.0	0.0
Morphine 200 mg granules capsule	0.0	0.0	0.0
Morphine 10 mg/mL injection	0.0	0.0	0.0
Morphine 10 mg/5 mL oral liquid	0.0	0.0	0.0
Vitamin A 100000 IU capsule	60.0	3.2	17.1
Vitamin A 200000 IU injection	10.0	0.0	2.4
Paracetamol 100 mg tablet	80.0	58.0	63.4
Paracetamol 120 mg/5 mL oral liquid	80.0	74.0	75.6
Paracetamol 200 mg suppository	80.0	93.5	90.2
Oxygen medicinal gas	0.0	0.0	0.0
Procaine benzyl penicillin 1 g powder for injection	70.0	51.6	56.1
Artemisinin combination therapy tablet	60.0	19.4	29.3
Artesunate 50–200 mg injection	40.0	12.9	19.5
Artesunate 50–200 mg suppository	0.0	0.0	0.0
Zinc 20 mg tablet	90.0	80.6	82.9
ORS, in sachets	90.0	96.8	95.1
Ampicillin 250 mg powder for injection	0.0	0.0	0.0
Ampicillin 500 mg powder for injection	60.0	9.7	22.0
Ampicillin 1 g powder for injection	0.0	0.0	0.0

Availability data on priority life-saving medicines used for the treatment of pneumonia, malaria and diarrhea are presented in Table 2. For the treatment of diarrhea, 90% of the public and 97% of the private sectors had ORS, whereas zinc was available in 90% of the public health facilities and 80% of the private sectors. Anti-malarial formulations of artemisinin combination therapy tablet and artesunate injection were available in 60 and 40% of public and 20 and 13% of private sectors, respectively. Rectal artesunate was not available in all surveyed medicine outlets. Regarding the availability of medicines for pneumonia, amoxicillin, 250 and 500 mg capsules were available in almost all sectors. Gentamycin injection was obtained in 90% of the public and 70% of the private sectors. Likewise, ceftriaxone 250 mg powder for injection was present in all public sectors and 7% of the private sectors. Ampicillin 250 mg and 1 g powder for injections were absent in all private and public sectors. However, ampicillin 500 mg powder for injections was found in 60% of the public and 10% of the private sectors.

**Table 2:** Availability of priority life-saving medicines used for the treatment of pneumonia, malaria and diarrhea for children under-five in selected health sectors found in Northern Ethiopia, 2016

Illness	WHO recommended priority life-saving medicine and their dosage forms	Percentage (%) of medicine outlets where medicine was found		
		Public sector (10 outlets)	Private sectors (31 outlets)	Overall (41 outlets)
Pneumonia	Amoxicillin 250 mg capsule	100.0	93.5	95.1
	Amoxicillin 500 mg capsule	100.0	100.0	100.0
	Ampicillin 250 mg powder for injection	0.0	0.0	0.0
	Ampicillin 500 mg powder for injection	60.0	9.7	22.0
	Ampicillin 1 g powder for injection	0.0	0.0	0.0
	Ceftriaxone 250 mg powder for injection	100.0	6.5	9.8
	Ceftriaxone 1 g powder for injection	100.0	81.0	85.4
	Gentamicin 40 mg/mL injection	90.0	71.0	75.6
	Oxygen medicinal gas	0.0	0.0	0.0
Malaria	Artemisinin combination therapy tablet	60.0	19.4	29.3
	Artesunate 50–200 mg injection	40.0	12.9	19.5
	Artesunate 50–200 mg suppository	0.0	0.0	0.0
Diarrhea	Zinc 20 mg tablet	90.0	80.6	82.9
	ORS, in sachets	90.0	96.8	95.1

### Patient’s price of priority life-saving medicines

In general, the medicines in this survey were sold at higher prices than the IRP (Table 3). The MPRs of priority life-saving medicines for the public and private sectors ranged from (0.2–19.4) and (0.4–26.2), respectively. The median MPRs of 10 LPGs for public were 1.5 times the IRPs, while the median MPRs for LPGs in private were 2.7 times the IRPs. Moreover, only one medication from each sector was bought at lower prices than its IRP. On contrary, five medicines in public sector and seven medicines in private sector were more than twice of their IRPs (Table 3).

**Table 3:** Median price ratios of ten lowest priced medicines for children under-five years of age found in at least four medicine outlets in public and private sectors in Northern Ethiopia, 2016

Name of the Medicine	Lowest priced medicines (MPR)	
	Public sector (25th–75th percentile)	Private sector (25th–75th percentile)
Amoxicillin 250 mg capsule	1.1 (1.1–1.2)	1.6 (1.1–1.8)
Amoxicillin 500 mg capsule	1.5 (1.4–1.5)	1.8 (1.5–1.8)
Gentamicin 40 mg/mL ampoule	1.1 (0.9–1.7)	2.0 (1.3–2.9)
Ceftriaxone 1 g vial	1.2 (1.2–1.2)	2.7 (2.2–2.7)
Paracetamol 100 mg tablet	3.4 (2.3–4.6)	4.6 (4.6–6.9)
Paracetamol 120 mg/5 mL suspension	19.4 (17.9–23.4)	26.2 (21.8–32.7)
Paracetamol 125 mg suppository	0.2 (0.2–0.2)	0.4 (0.4–0.4)
Penicillin G 1 million IU vial	1.5 (1.3–1.6)	2.7 (2.5–3.1)
ORS to make 1000 mL solution	3.7 (2.4–3.8)	4.6 (3.7–4.6)
Ampicillin 500 mg Vial	8.2 (7.2–11.5)	13.7 (5.6–20.6)

Among the priority life-saving medications, the lowest MPRs was observed for paracetamol 125 mg suppository at both public (0.2) and private (0.4) sectors, as opposed to paracetamol oral suspension with MPRs of 19.4 in public and 26.2 in private sectors (Table 3). Besides, the highest price difference between the two sectors was noted in paracetamol suspension, which was 25% more in the private sector than the public sector.

### Treatment affordability for selected disease conditions with priority life-saving medicines

The affordability of standard treatments for six different health conditions (10 medicines) is described in Table 4. About 30% of priority life-saving medicines in public sector and 50% of them in private sector required more than a single daily wage to purchase the standard treatment of the prevalent diseases of children. The wages required to purchase the standard treatment of LPGs for public and private sectors were in the ranges of 0.2 (ORS and paracetamol tablet) to 8.0 (penicillin G 1MIU) and 0.2 (paracetamol tablet) to 14.1(penicillin G 1MIU), respectively. Some treatments were very costly (Table 4).

**Table 4:** Number of days’ wage required for the lowest paid Ethiopian government worker to purchase standard treatment for children under five of age in Northern Ethiopia, 2016

			Days wages to pay for treatment		
			Public sector	Private sector	Private to public ratio
Non-severe pneumonia	Ampicillin 500 mg vial	50 mg/kg*14.5 kg QID IV for 5 days = 14,500 mg = 29 vial	4.8	8.1	1.7
Severe pneumonia	Amoxicillin 250 mg capsule	25 mg/kg*14.5 kg BID P.O. for 7 days = 5075 mg = 21cap	0.5	0.7	1.4
Severe pneumonia	Amoxicillin 500 mg capsule	25 mg/kg*14.5 kg BID P.O. for 7 days = 5075 mg = 11 cap	0.5	0.6	1.2
Severe pneumonia	Penicillin G 1000,000 IU vial	50,000 units/kg*14.5 kg IV every 4 h for at least 3 days = 13.03 millions of IU = 14 vial	3.9	6.8	1.7
Very severe pneumonia	Gentamicin 80 mg/2 mL ampoule	7.5 mg/kg*14.5 kg IV daily for 5 days = 14 ml 7 ampoule	0.6	1.1	1.8
Very severe pneumonia	Ceftiaxone 1 g vial	80 mg/kg*14.5 kg IV daily for 10 days = 11.6 g 12 vial	6.1	13.9	2.3
Neonatal sepsis	Gentamicin 80 mg/2 mL (40/mL) ampoule	5 mg/kg*14.5 kg IV daily for 10 days = 20 ml 10 ampoule	0.9	1.6	1.8
Neonatal sepsis	Penicillin G 1000,000 IU vial	50,000 units/kg*14.5 kg IV QID for 10 days = 29 millions of IU = 29 vial	8.0	14.1	1.8
Dehydration	Oral rehydration salt, in sachets	75 ml/kg*14.5 kg = 1087.5 ml	0.2	0.3	1.5
Pain/inflammation	Paracetamol 125 mg/5 mL suspension	5 years old child: P.O. 15 mg/kg*14.5 kg*4*3 = 104.4 ml	0.4	0.6	1.5
Pain/inflammation	Paracetamol 125 mg suppository	5 years old child: rectal 15 mg/kg*14.5 kg*4*3 = 20.88 = 21 suppositories	1.0	1.9	1.9
Pain/inflammation	Paracetamol 100 mg tablet	5 years old child: P.O. 15 mg/kg*14.5 kg*4*3 = 26.1 = 27 tabs	0.2	0.2	1.0

The highest ratio between the private and public sectors was observed in ceftriaxone 1 g vial, which was used for the management of very sever pneumonia. Overall, when the affordabilities in public and private sectors were compared, priority life-saving medicines in private sectors were less affordable than public sectors (Table 4).

**Fig. 2:**
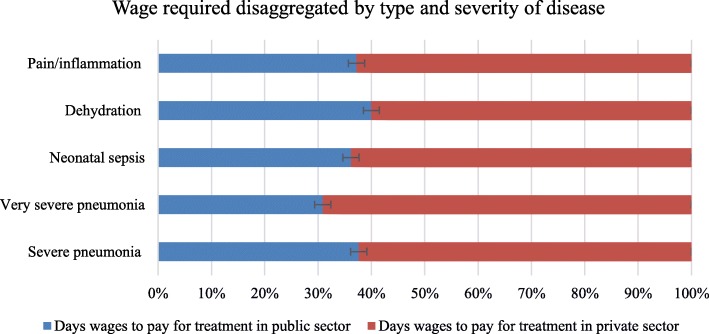
Number of days’ wage required for the lowest paid Ethiopian government worker. Blue colored portions of the graphs represent daily wages required to cover the total costs for full course of therapy of the selected diseases in public sectors. Red colored portions of the graphs represent daily wages required to cover the total costs for full course of therapy of the selected diseases in public sectors. Legend: The horizontal bar that intersects both sectors denotes an error bar

Figure 2 shows a stacked bar chart – used to compare the percentage that each value contributes to a total – that compares the percentage of wages attributable to the private and public sector. Accordingly, the share contributed by the public sector was below 40% for all disease conditions, in which about one third of the wage for very severe pneumonia was attributable to the public sector.

## Discussions

According to the findings of this study, the overall availability of priority life-saving medicines was found to be low. Due to variation in medicine pricing policy, methodology, types of prevalent disease, and medicine supply systems, it is difficult to make a comparative analysis of medicines availability. However, this result is in agreement with studies of the availability and affordability of essential medicines in Ethiopia [10] and elsewhere [18, 24, 25]. This calls for urgent action to address the availability of life-saving medications in the region.

The average availability of lowest priced medicines for children was 41.9% in the public and 31.5% in the private sectors. These findings are lower than a study done in Western part of Ethiopia [10] which reported 43% for public and 42.8% for private sectors; comparable with reports of a study conducted in Guatemala [22] which found an availability 46% in public sector and 35% in private sector whereas higher than the study done by Wang et al. 2014 [18] in China, which reported availability of 27.3% for public sector and 20.6% for private sector. In agreement with the studies done elsewhere [18, 22], the present study showed that availability of medicines was higher in the public sector than in the private sector. However, considering the particular health service needing population, still, the figure in public sector was very low. The low availability of medicines at public hospitals could have direct implications on access, as patients are then persuaded to purchase these medicines from private pharmacies where quite often are sold for higher price. Private pharmacies most of the time carry fewer generic drugs than the public sectors; as a result, they may dispense more brand medicines. Obviously, brands are more costly than their generic equivalents that lead the patients to dig deep into their pockets to pay for medicines [26].

It was noted in this study that 11 medicines out of the 27surveyed priority life-saving medicines were absent in both private and public sectors. Particular concerning is the unavailability of rectal artesunate, ampicillin (250 mg and 1 g) injection and medicinal oxygen, which are the WHO recommended life-saving priority medicines for the treatment of malaria and pneumonia, even though lower respiratory tract infections including pneumonia and malaria are the foremost causes of death and disease burden among under-five children in Ethiopia [27, 28]. This finding is in line with a study done in Uganda [29]. According to some of the administration of the medicine outlets, the possible reason for the lower availability of ampicillin formulations in this study was due to the choice of ceftriaxone injection for many conditions over ampicillin. In addition to ampicillin and artesunate, all of the morphine dosage forms for palliative care and pain management and medicinal oxygen gas for pneumonia were absent in this study. The observed lack of availability of morphine could be partly tied to pethidine, which is the first line for pain management as compared to morphine, which is listed as the alternative treatments for pain management in the country’s standard treatment guideline [19].

Despite its known clinical benefit and proven effectiveness in reducing mortality from pneumonia [30], medicinal oxygen was found to be absent in almost all sectors surveyed in this study. This finding is very disquieting because oxygen is perhaps the only drug with no alternative agent [31]. Oxygen therapy should therefore be available for children care in every sector especially for the management of pneumonia as it is the leading cause of death in children under-5 worldwide.

The prices of priority life-saving medicines in this study were relatively higher as compared to IRPs. There was a variation in prices for medicines in public and private sectors. In the public sector, they were sold at 1.5 times their IRP and 2.7 times their IRP in the private sector. Similar findings were reported in another local study [10] as well as studies in South America [22] and Asia [18]. The reason for the lower price of medicines in public sectors could be the effort made by the Ethiopian government to reduce drug prices over the last decade by designing various mix of policies to regulate the price on pharmaceutical products so that there would not be higher price mark-ups in public sectors.

Despite the fact that it is difficult to assess true affordability, treatments costing one day’s wage or less are generally considered affordable. Assessed accordingly, in the current study, about 30% of medicines in public sector and 50% of them in private sector were unaffordable. This shows that a significant segment of the population would not be able to pay for their medicines. Even medicines like amoxicillin, paracetamol tablet and gentamicin which were seemed affordable for the lowest government wage could be out-of-reach for a substantial number of people in Ethiopia because around 30% of the population in the country is living below the international poverty line (defined as an income of less than $1.9/day) [32]. These costs do not even include the costs of consultation and diagnostic tests; hence, families who need medicines for more than one child may be confronted with more costs and extra days’ wages. These findings are consistent with other studies [10, 22, 33, 34] done on the affordability of essential medicines for children.

Eventhough Ethiopia achieved millennium development goal for reducing child mortality, the findings from this study advocate that availability and affordability of priority life-saving medicines for children is still low. High medicine prices and low incomes are considered as the notable barriers to the affordability of treatments particularly in developing countries. Country or regional health authorities must therefore improve the availability of more affordable generic priority life-saving medicines in the public sector by monitoring efficiency of the public sector procurement system as well as encouraging local pharmaceutical manufacturing. Besides, regulatory authorities need to provide a regulatory and enforcement mechanism in which cheaper alternative medicines would be more often prescribed, dispensed and used than newer, more expensive medicines. In general, this study suggests the regional priority life-saving medicine policy to be established, developed and enforced at both public and private sectors to ensure availability and affordability to basic health services, particularly for the poor.

This study has a certain limitation. It did not explore the factors affecting the availability and utilization of the priority medicines for children in public and private health facilities in Tigray region. Undertaking a more in-depth study to explore the underlying factors is needed. The study did not survey medicine procurement prices at wholesales due to logistical constraints. Percentage of medicines availability at the time of data collection may not be the same all year long. Since the study was predominantly based on WHO/HAI methodology, the concerns pertaining to the representativeness of the selected medicine outlets still can arise. Moreover, patient charges in all sectors were compared with IRPs, which do not consider freight and other margins and markups. This may affect the validity of the price comparison.

## Conclusions

This study divulges that the availability and affordability of life-saving priority medicines for children under-five were limited in health facilities of the region in spite of the WHO emphasis. This result calls for urgent action to address the availability of life-saving medications in the region. An integration of these life-saving medicines into the logistic and essential drug list of the health facilities remains pertinent to increase their availability and affordability.

## Abbreviations

BID: Two times a day
g: gram
IRP(s): International Reference Unit Price(s)
IU: International Unit
Kg: kilogram
LPG: Lowest Priced Generic
mg: milligram
mL: Milliliter
MPR(s): Median Price Ratios(s)
MSH: Management Science for Health
OB: Originator Brand
ORS: Oral Rehydration Salts
PO: per oral
QID: Four times a day
WHO/HAI: World Health Organization/Health Action International
